# The Contributions of Statistical Learning to L2 Morphosyntax Processing Across Visual and Auditory Modalities in Chinese Adolescent Learners

**DOI:** 10.3390/bs16081452

**Published:** 2026-08-21

**Authors:** Kaiyue Song, Weijia Yan, Guoying Yang, Yueyan Huang, Hui Yu, Li Li

**Affiliations:** 1 School of Foreign Studies, South China Normal University, Guangzhou 510631, China; 2022010106@m.scnu.edu.cn (K.S.); veegeeyan@m.scnu.edu.cn (W.Y.); yangguoying@m.scnu.edu.cn (G.Y.); 2024010125@m.scnu.edu.cn (Y.H.); 2 Bandao School, Huiyang District, Huizhou 516221, China; yuhuivicky2026@163.com; 3 Key Laboratory of Chinese Learning and International Promotion, School of International Culture, South China Normal University, Guangzhou 510631, China

**Keywords:** statistical learning, L2 morphosyntax processing, modality, adolescent, self-paced reading and listening

## Abstract

Individual differences in statistical learning (SL) constitute an important cognitive mechanism that supports second language (L2) syntactic processing. However, existing research has primarily focused on adult populations, while potential differences across input modalities remain understudied. This study investigated the associations between SL and implicit L2 morphosyntactic processing among Chinese adolescent English learners, comparing results across visual and auditory modalities. Participants completed visual and auditory SL tasks, self-paced reading (SPR), self-paced listening (SPL), as well as working memory (WM) and attention tasks. L2 morphosyntactic processing ability was measured via grammatical sensitivity indicators extracted from SPR and SPL data. Mixed-effects analyses did not detect statistically reliable group-level grammaticality effects in either task, though SPR exhibited a descriptive pattern where ungrammatical sentences elicited longer reading times at the third word. Participants achieved higher accuracy on the visual SL task than the auditory one. In exploratory regression analyses, visual SL accuracy was found to positively predict grammatical sensitivity measured at Word 3 in SPR. Still, this association was not confirmed in a follow-up sensitivity analysis. No corresponding significant association was observed between auditory SL and SPL sensitivity, and neither working memory nor attention showed consistent independent contributions to the outcome. These findings extend individual-differences research on this topic to adolescent classroom L2 learners and underscore the potentially important role of visual regularity learning in online L2 reading processing.

## 1. Introduction

Morphosyntax often encodes subtle grammatical information that is not essential for sentence comprehension, making it particularly vulnerable in second language (L2) processing. This processing difficulty caused by low salience is especially evident among learners whose first language (L1) lacks overt inflectional morphology, such as Mandarin Chinese (White, 2003). Previous research on Chinese learners of English as a foreign language (EFL), ranging from child learners to advanced adult learners, has largely attributed these processing difficulties to individual differences in executive functions, including working memory (WM) and attention (e.g., Just & Carpenter, 1992; Coughlin & Tremblay, 2013; McDonald, 2006; McDonough & Trofimovich, 2016; Novick et al., 2005, 2010). More recently, statistical learning (SL)—the ability to extract regularities from environmental input—has been shown to support both L1 (e.g., Kidd, 2012; Kidd & Arciuli, 2016; Misyak, 2010; Misyak & Christiansen, 2012) and L2 morphosyntax processing (e.g., Ettlinger et al., 2016; Godfroid & Kim, 2021; Granena, 2013; Lee, 2023; McDonough & Trofimovich, 2016; Suzuki et al., 2023; Pili-Moss et al., 2025). However, growing evidence suggests that SL may be modality-specific. For example, Conway and Christiansen (2005) found that speakers of alphabetic languages show an advantage in auditory statistical learning (ASL), while studies conducted on Chinese populations have demonstrated a visual statistical learning (VSL) advantage (He & Tong, 2017; Tong et al., 2019). This pattern indicates that L1 writing system experience may shape modality-specific SL mechanisms, which in turn may influence the relationship between SL and L2 morphosyntax processing.

Input modality has also been identified as an important factor in L2 morphosyntax processing (K. M. Kim & Godfroid, 2019). A growing body of research has examined morphosyntax sensitivity under visual and auditory conditions separately (e.g., Jiang, 2004, 2007; Godfroid & Kim, 2021; Faretta-Stutenberg & Morgan-Short, 2018; Song, 2015). However, studies directly comparing modalities have produced mixed results, with evidence for both visual (K. M. Kim & Godfroid, 2019; Zhuang et al., 2025) and auditory (Meulman et al., 2014; Zhao et al., 2021) advantages. Notably, Zhuang et al. (2025) found that high-proficiency Chinese EFL learners demonstrated sensitivity to the progressive *-ing* inflection only under visual input conditions, indicating a visual processing advantage in Chinese L2 learners’ syntactic processing.

Taken together, these findings suggest that modalities may modulate both SL and L2 morphosyntax processing, which may further influence the relationship between SL and L2 morphosyntax processing. For example, Qi et al. (2019) reported that, compared with VSL, ASL provides greater support for alphabetic-language speakers’ reading literacy. However, two limitations remain in the literature: (1) the role of modality in the relationship between SL and L2 morphosyntax processing has not been systematically examined in Chinese EFL learners, and (2) most existing studies focus on adult learners. Given that adolescent learners are in a critical developmental stage of both cognitive and L2 acquisition processes, investigating this population may provide a more sensitive test of SL–morphosyntax relationships. Therefore, the present study examines the relationship between SL and L2 morphosyntax processing across visual and auditory modalities in Chinese adolescent learners.

### 1.1. Visual and Auditory L2 Morphosyntax Processing

Whether visual or auditory input better facilitates implicit L2 learning has not been thoroughly studied in L2 research (Zhao et al., 2021). According to the attentional hypothesis (Schmidt, 2012), understanding spoken input involves speech decoding, which places a heavy load on cognitive and attentional resources. By contrast, written language provides relatively stable orthographic and morphological information and generally allows learners greater control over their processing pace. These differences may influence learners’ sensitivity to low-salience morphosyntactic forms.

Previous studies have produced mixed evidence regarding modality-related differences in L2 grammatical learning and processing. Studies using training, behavioral, and neurophysiological paradigms have sometimes reported learning advantages following written input. For instance, grammatical structure learning in semi-artificial and natural language contexts is more sustained with written input (K. M. Kim & Godfroid, 2019; Zalbidea, 2021). Additionally, highly proficient Chinese EFL learners exhibit stronger neurophysiological sensitivity to inflectional violations when processing written input (Zhuang et al., 2025). However, other studies reported comparable learning across modalities or an auditory advantage under particular conditions. Coumel et al. (2023), for instance, observed similar longer-term syntactic learning effects across visual and auditory conditions, although short-term learning was more sensitive to auditory input.

These mixed findings may reflect differences in learner proficiency, target structures, exposure conditions, and outcome measures and therefore do not establish a general advantage for either visual or auditory input. To examine grammatical sensitivity across input modalities under more closely aligned conditions, the present study administered self-paced reading (SPR; Godfroid & Kim, 2021; Jiang, 2004, 2007; Song, 2015) and self-paced listening (SPL; J. Kim et al., 2020; Papadopoulou et al., 2013) tasks using the same sentence materials with the same group of participants. This design enabled reading- and listening-based grammatical sensitivity to be examined under closely aligned linguistic conditions.

### 1.2. Modality-Specific Mechanism of Statistical Learning

SL performance may not be fully shared across sensory modalities (e.g., Conway & Christiansen, 2005; Hu et al., 2026). In a direct comparison of visual and auditory triplet learning, Qi et al. (2019) found no significant individual-level association between performance in the two modalities, suggesting that sensitivity to regularities in auditory input may not correspond closely to sensitivity in visual input. Auditory advantages have been observed in sequence- and artificial grammar learning tasks involving participants with alphabetic L1 backgrounds (Conway & Christiansen, 2005; Lukics & Lukács, 2022).

However, this pattern has not been observed consistently across learner populations. Ren and Wang (2023) found that Chinese-speaking children performed above chance in both visual and auditory SL tasks but showed stronger performance in some visual conditions. Taken together, these findings suggest that VSL and ASL may involve partly distinct learning abilities and that their relative strength may vary across populations and task conditions. One possible source of this variation is learners’ prior language experience, which is considered in the following section.

### 1.3. Contribution of Statistical Learning to L2 Morphosyntax Processing

The “linguistic entrenchment” hypothesis proposes that learners’ prior experience with linguistic structures shapes their expectations about co-occurrence patterns and thereby influences their sensitivity to statistical regularities (Siegelman et al., 2018). Consistent with this possibility, some studies involving children and adults with alphabetic L1 backgrounds have found associations between ASL and phonological processing, morphological knowledge, or reading-related abilities (e.g., Spencer et al., 2015; Qi et al., 2019; Lukács et al., 2023; Ren et al., 2023). Studies of Mandarin-speaking learners have instead reported associations between VSL and Chinese orthographic awareness or literacy-related abilities (He & Tong, 2017; Tong et al., 2019), as well as stronger performance in some visual than auditory SL conditions (Ren & Wang, 2023). These cross-study patterns suggest that language and literacy experience may be related to modality-based variation in SL. However, because the studies differed in their participants, stimuli, tasks, and outcome measures, they do not demonstrate that alphabetic and logographic L1 backgrounds necessarily lead to auditory and visual SL advantages, respectively.

Previous individual-differences studies have also examined associations between SL and L2 morphosyntactic learning, knowledge, or online processing using a range of learner populations and outcome measures (see Supplementary File S1). Much of this evidence comes from adult learners, particularly Chinese-L1 learners of Japanese or English in immersion contexts.

Sequence-learning measures have been associated with some agreement, particle, definiteness, and timed accuracy outcomes, but the observed associations have varied across target structures and language-processing tasks. Evidence from younger instructed learners remains comparatively limited. Pili-Moss et al. (2025) found that SL interacted with WM in predicting English grammar development and written-task outcomes in German-speaking adolescent EFL learners. However, that study examined learning and production outcomes rather than incremental sensitivity to morphosyntactic violations during online sentence comprehension. It therefore remains unclear whether SL is associated with adolescent learners’ online L2 morphosyntax processing and whether the association patterns of differ across modalities. The present study addresses this gap by assessing visual and auditory SL together with SPR and SPL in the same group of Chinese adolescent EFL learners.

### 1.4. Working Memory and Attention as Cognitive Covariates

Working memory (WM) and executive attention may contribute to individual differences in L2 morphosyntax processing. WM supports the temporary maintenance and manipulation of linguistic information (Just & Carpenter, 1992) and has been associated with L2 grammatical sensitivity, particularly for agreement structures and under demanding processing conditions (Coughlin & Tremblay, 2013; McDonald, 2006; McDonough & Trofimovich, 2016; Zalbidea & Sanz, 2020). Recent evidence further suggests that WM may operate jointly with SL in supporting morphosyntactic learning and performance (Pili-Moss et al., 2025).

Executive attention may facilitate the detection and resolution of competing linguistic representations during comprehension and production (Novick et al., 2005, 2010). Previous studies have associated cognitive control with sensitivity to syntactic violations and agreement errors (Veenstra et al., 2018; Ye & Zhou, 2009). Because WM and executive attention may be related to both SL performance and L2 morphosyntax processing, they were included as cognitive covariates in the present study.

### 1.5. The Present Study

The present study addresses two research questions: (1) What patterns of association emerge across visual and auditory SL and reading- and listening-based morphosyntactic processing? (2) Do VSL and ASL predict implicit L2 morphosyntactic processing after WM and attention are controlled? Given previously reported visual advantages in SL among Mandarin-speaking learners and the prominent role of written input in Chinese EFL instruction, we hypothesized that higher VSL performance would be associated with greater L2 morphosyntactic sensitivity in visual input. Because previous evidence concerning ASL was less consistent, its relationship with L2 morphosyntax processing was examined exploratorily.

## 2. Materials and Methods

### 2.1. Participants

Participants were Chinese adolescent EFL learners recruited from three first-year junior secondary classes at the same school in South China. Midterm English examination scores were used during recruitment to ensure that participants had sufficient English proficiency to complete the language-processing tasks. An a priori power analysis was conducted in G*Power (Version 3.1.9.7) for the planned two-tailed bivariate correlation between VSL accuracy and the Grammatical Sensitivity Index (GSI), with α = 0.05 and power = 0.80. Based on the effect size reported by Godfroid and Kim (2021; r = 0.341), the required sample size was 63. A total of 80 students initially participated. One student was excluded because parental informed consent was unavailable, leaving 79 eligible participants. Because of equipment failure, absence from individual testing sessions, and incomplete task data, the valid sample size varied across tasks and analyses. Valid sample sizes for each task and analysis were reported in Section 3.2.

Among the 74 participants with available examination scores, the mean score was 81.85 (SD = 7.94, range = 76–96). Midterm English scores were also included as a covariate in the regression analyses to account for individual differences in English proficiency. The participants followed the same school curriculum and had broadly comparable classroom-based English learning experience. Parent-reported background-questionnaire data were available for subsets of the sample because some responses were incomplete. Among participants with valid demographic data, the mean age was 13.25 years (SD = 0.56, range = 12–14; N = 64), and 21 were male and 45 were female (N = 66). Participants began formal English instruction at a mean age of 7.23 years (SD = 1.80; N = 64) and had studied English for an average of 6.68 years (SD = 1.77; N = 65). None of the 61 participants with valid responses reported having lived in an English-speaking country. Detailed demographic and language-background information is provided in Supplementary File S2.

### 2.2. Tasks

The present study used a within-participant, cross-modal design. The same participants completed visual and auditory SL tasks together with reading- and listening-based morphosyntactic processing tasks, and the SPR and SPL tasks used the same sentence materials. This design was suited to examining whether SL–morphosyntax association patterns differed across visual and auditory tasks while reducing variation attributable to different participant samples and linguistic materials.

#### 2.2.1. Tasks for Implicit L2 Morphosyntax Processing

Materials: Three target morphosyntactic structures were selected to assess L2 morphosyntactic processing: (1) third-person singular agreement, (2) plural marking, and (3) past-tense marking (see Table 1 for examples). These structures represent different stages in the development of English inflectional morphology (Ellis, 2008) and have been widely used in L2 research because of their well-documented acquisition difficulty.

The original materials comprised 48 target items, each with a grammatical and an ungrammatical version, yielding 96 critical-sentence versions. The two versions of each item were distributed across participants using two counterbalanced lists (J. Kim et al., 2020; Suzuki & Sunada, 2018). Twenty-four filler sentences matched approximately for length and structural complexity were also included.

Following data collection, the materials underwent a blinded post hoc linguistic audit by two independent evaluators with expertise in English experimental psycholinguistics. For each critical item, the evaluators assessed whether (a) the grammatical version was fully grammatical and natural, (b) the ungrammatical version was unambiguously ungrammatical, (c) the intended violation was confined to the predefined critical region, and (d) the two versions were equivalent in meaning and structure apart from the target manipulation. The comprehension questions were additionally evaluated for grammaticality, answerability, consistency with the sentence meaning, and the validity of the designated correct response. All criteria were rated as Yes, No, or Ambiguous. Evaluators were required to explain every Ambiguous judgment. Any item receiving at least one Ambiguous judgment from either evaluator was retained in the primary analysis but excluded in the sensitivity analysis.

The audit identified five items with problems that could invalidate the intended experimental contrast. Both counterbalanced versions of these items were excluded, leaving 43 target items, corresponding to 86 critical-sentence versions. The original experimental materials and the item-level procedures and decisions of the post hoc linguistic audit are provided in Supplementary File S3. An additional 10 items contained less severe grammatical, spelling, or comprehension-question problems that did not directly compromise the predefined critical or spillover regions. These items were retained in the primary analyses but excluded in the sensitivity analyses, the results of which are reported in Supplementary File S4.

Target-word frequency was also obtained from SUBTLEX-US and expressed on the Zipf scale. After exclusion of the incorrectly classified items, frequency did not differ significantly between the grammatical items (M = 4.37, SD = 0.91) and the ungrammatical items (M = 4.59, SD = 0.90), Welch’s *t*(44.97) = −0.82, *p* = 0.419. Target-word length also did not differ significantly between the grammatical items (M = 5.75, SD = 1.62) and the ungrammatical items (M = 5.70, SD = 2.20), Welch’s *t*(40.36) = 0.10, *p* = 0.924.

Self-Paced Reading Task for Visual Input: In the SPR task, sentences were presented word by word. Each word appeared centrally on a white background in Courier New (34-point, bold) font, and participants advanced through the sentence by pressing a key. Each new word replaced the previous one, and this process continued until sentence completion. Participants then proceeded either to the next sentence or answered a comprehension question to ensure attention to meaning.

Processing was indexed using reaction time (RT) at three regions: the critical region (the word containing the violation, hereafter Word 1) and two subsequent spillover regions (Word 2 and Word 3). This region-based analysis was adopted because processing difficulty triggered by a critical word may spill over to subsequent words, as eye movements or button-press responses can precede the completion of cognitive processing (Jegerski, 2013; Keating, 2009).

In addition, GSI was computed by subtracting reading times for grammatical sentences from those for ungrammatical sentences (ungrammatical—grammatical; Jiang, 2004). Positive values indicate increased processing cost for violations. This measure captures individual differences in sensitivity even when effects are not uniformly observable across regions (Godfroid & Kim, 2021; Granena, 2013; Suzuki & DeKeyser, 2017).

Self-Paced Listening Task for Auditory Input: To minimize variation attributable to differences in linguistic materials, the same sentence materials were used in an auditory SPL task. Sentences were recorded by a female native speaker of American English in a sound-attenuated environment and digitized at 44.1 kHz with 16-bit resolution. Each sentence was segmented into individual word files based on natural pause boundaries.

GSI was computed using the same logic as in SPR. However, RT for each word was calculated by subtracting the word’s acoustic duration from the interval between word offset and participant response, following established procedures for auditory self-paced paradigms (Papadopoulou et al., 2013).

#### 2.2.2. Tasks for Statistical Learning

Visual Statistical Learning: The VSL and ASL tasks were adapted from Schneider et al. (2020). In the VSL task, participants were exposed to a continuous stream of 12 capital letters. The letters were structured into four fixed triplets (GJA, FKC, LBE, MDH), which were unknown to participants. The letters were presented sequentially for 800 ms each, with a 200 ms interstimulus interval. The four triplets were repeated 24 times (96 triplets in total; exposure duration ≈ 4.48 min). A target-detection task was used to maintain attention during the exposure, in which one triplet was randomly selected, and its third letter served as the target stimulus. Participants were instructed to respond when the target letter appeared in the stream. After exposure, participants completed a two-alternative forced-choice (2AFC) test consisting of 32 trials. In each trial, one learned triplet was paired with a foil triplet (GDE, FJH, LKA, MBC), and participants selected the familiar triplet.

Accuracy (ACC) in the 2AFC was used as an offline measure of SL. VSL accuracy was calculated as the proportion of correct responses across the 32 forced-choice test trials. Internal consistency across the dichotomously scored test trials was acceptable (Cronbach’s α = 0.74; N = 72). Online SL was indexed by RT slope in the target-detection task, calculated as the change in RT across the exposure phase, with decreasing RT reflecting increased sensitivity to the embedded transitional regularities.

Auditory Statistical Learning: In the ASL task, participants were exposed to a continuous stream of 12 English syllables (pa, bi, ku, go, la, tu, da, ro, pi, ti, bu, do), structured into four fixed triplets (pabiku, golatu, daropi, tibudo), which were unknown to participants. The syllables were presented sequentially for 460 ms each, with a 20 ms interstimulus interval. The four triplets were repeated 48 times. To accommodate modality-specific differences in perceptual and temporal processing, the ASL task used a faster presentation rate and more triplet repetitions than the VSL task (Qi et al., 2019). A target-detection task was used to maintain attention, in which one triplet was randomly selected, and its third syllable served as the target stimulus. Participants responded whenever the target syllable appeared in the stream. After exposure, participants completed a 32-trial 2AFC test. In each trial, a learned triplet was paired with a foil triplet (parodo, gobuku, dabitu, tilapi), and participants selected the familiar sequence.

ASL was indexed using 2AFC accuracy and the RT slope from the target-detection task. Internal consistency across the dichotomously scored test trials was Cronbach’s α = 0.48 (N = 68). Although the ASL accuracy measure showed relatively low internal consistency, the task was retained because it was based on an established SL paradigm (Chen et al., 2023; Chen et al., 2026; Schneider et al., 2020) and provided an auditory counterpart to the parallel VSL task, which was necessary for examining modality-related association patterns. Nevertheless, the limited reliability may have attenuated associations involving ASL accuracy.

#### 2.2.3. Tasks for Working Memory and Attention

Working memory capacity was assessed using the operation span (OSPAN) task (Unsworth et al., 2005). In this task, participants sequentially solved simple math operations while memorizing a series of letters. After each trial, they were required to recall the letters in the correct order from a response array. The task included practice sessions for letter recall, math operations, and the combined dual-task procedure. The formal test consisted of 75 trials with set sizes ranging from 3 to 7 letters. Participants were required to maintain at least 85% accuracy on the math component to ensure valid data. The OSPAN score was calculated as the number of correctly recalled letters from perfectly recalled sets, serving as the index of WM capacity. The task was computer-based and typically lasted approximately 15 min.

Attention was measured using the Attention Network Test (Fan et al., 2002), which assesses three components: alerting, orienting, and executive control. Participants performed a flanker task in which they identified the direction of a central arrow while ignoring congruent or incongruent flanking arrows. Different cue conditions (no cue, center cue, double cue, and spatial cue) were presented before target onset. Alerting was computed as the RT difference between the no-cue and double-cue conditions. The difference between center-cue and spatial-cue conditions indexed orienting. Executive control was measured as the RT difference between incongruent and congruent flanker conditions.

### 2.3. Procedures

The experiment was administered across three sessions to reduce fatigue and potential carry-over effects. Session 1 included the VSL task, followed by SPR and ASL tasks. Session 2 included WM and the attention task. Session 3 included SPL and the grammatical judgment test.

This arrangement served two purposes. First, distributing tasks across sessions reduced cognitive load, particularly for adolescent participants completing a relatively long test battery. Second, SPR and SPL were separated by approximately three weeks to minimize potential practice or material-specific transfer effects between visual and auditory morphosyntax processing tasks.

### 2.4. Data Preprocessing and Analysis

For both SPR and SPL, trials associated with incorrect comprehension responses were first excluded separately for each participant (SPR: 556 trials, 15.24%; SPL: 534 trials, 15.89%). Participant-specific means and standard deviations were subsequently calculated separately for each word position, and RTs exceeding ±3 SD from the corresponding mean were removed. This excluded a further 103, 105, and 103 word-region observations at Words 1, 2, and 3, respectively, in SPR and 40, 40, and 36 observations, respectively, in SPL.

Following the linguistic audit, exclusion of the five invalid items removed 283 sentence trials in SPR and 264 sentence trials in SPL. The resulting primary-analysis datasets included 43 items and 7895 word-region observations in SPR and 43 items and 7437 word-region observations in SPL. The sensitivity analyses additionally excluded the 10 items with less severe linguistic or comprehension-question problems, removing a further 497 SPR trials and 493 SPL trials. Thus, relative to the original 48-item set, the sensitivity analyses excluded 15 items and a total of 780 sentence trials in SPR and 757 sentence trials in SPL.

Following preprocessing, word-level RTs were log-transformed before analysis. Linear mixed-effects models were fitted separately for SPR and SPL in R version 4.6.1, using the lme4 package. Models were estimated using restricted maximum likelihood. Fixed-effect tests were obtained using the lmerTest package (version 3.2-1), with degrees of freedom and two-sided *p* values calculated using the Satterthwaite approximation. Model-based comparisons were conducted using the emmeans package (version 2.0.4).

Grammaticality, word position, and their interaction were included as fixed effects. Both predictors were coded. Grammatical sentences served as the reference level for grammaticality (Gram = 0, Ungram = 1), and Word 1 served as the reference level for word position, with separate contrasts comparing Word 2 and Word 3 with Word 1. For each task, the initial model included random intercepts and uncorrelated random slopes for grammaticality, the two word-position contrasts, and their interactions at both the participant and item levels. Random-effect correlations were fixed at zero. Model singularity was evaluated using lme4::isSingular() with a tolerance of 10^−4^. When a model was singular, the random-effects structure was simplified sequentially while the fixed-effects structure was kept unchanged.

For SPR, the participant- and item-level interaction slopes were removed first because their estimated variances were zero or effectively zero. The resulting model remained singular because the participant-level Word 2 slope had an estimated variance of zero. After this slope was removed, the model was no longer singular. The final SPR model retained participant-level slopes for grammaticality and Word 3 and item-level slopes for grammaticality, Word 2, and Word 3.

For SPL, the participant- and item-level interaction slopes were first removed because their estimated variances were zero or effectively zero. The model remained singular, and the participant-level Word 2 and Word 3 slopes, both of which had zero estimated variance, were removed. The item-level Word 2 and Word 3 slopes were then removed sequentially because their estimated variances remained zero. The final SPL model retained grammaticality slopes for both participants and items. Both final models converged successfully and did not produce singular fits. Model syntax, variance estimates for each simplification step, and software session information are provided in Supplementary File S5.

No additional preprocessing was applied to the SL tasks. WM data were retained for all participants, as accuracy on the secondary math task exceeded the validity threshold of an accuracy rate of 85% (M = 92.94%). For the attention task, incorrect trials (3.6%) were excluded, and RTs exceeding ±3 SD from the mean were removed (17.7%). The remaining trials were used to compute attention network scores for subsequent analyses.

Multiple imputation was not applied because missingness primarily involved unavailable entire task datasets rather than isolated values within otherwise complete records. Task-specific analyses included all participants with valid data for the relevant task. The linear mixed-effects models used all retained trial-level observations following the preprocessing procedures. Correlation analyses were based on pairwise complete observations, whereas the hierarchical regression analyses used complete cases for the outcome and all predictors included in the respective model. Accordingly, analytic sample sizes varied across tasks and analyses and are reported explicitly in Section 3.2.

## 3. Results

### 3.1. Implicit L2 Morphosyntax Processing Results

The final SPR model retained participant-level slopes for grammaticality and Word 3 and item-level slopes for grammaticality, Word 2, and Word 3. As reported in Table 2, the main effect of grammaticality at Word 1 was not significant, *β* = 0.008, SE = 0.016, *t* = 0.48, *p* = 0.635. RTs at Word 2 were longer than those at Word 1, *β* = 0.029, SE = 0.014, *t* = 1.98, *p* = 0.050, whereas RTs at Word 3 were significantly longer than those at Word 1, *β* = 0.083, SE = 0.019, *t* = 4.40, *p* < 0.001. Neither the Grammaticality × Word 2 interaction, *β* = 0.001, SE = 0.018, *t* = 0.08, *p* = 0.935, nor the Grammaticality × Word 3 interaction, *β* = 0.032, SE = 0.018, *t* = 1.73, *p* = 0.084 was significant. Model-based comparisons showed no significant grammaticality differences at Word 1 or Word 2, *p*s ≥ 0.572. At Word 3, RTs were longer for ungrammatical sentences (*M* = 699.58 ms, SD = 220.13) than for grammatical sentences (M = 670.38 ms, SD = 204.71), *β* = 0.039, SE = 0.016, *t* = 2.39, *p* = 0.018.

The final SPL model retained grammaticality slopes for both participants and items. As reported in Table 3, the main effect of grammaticality at Word 1 was not significant, *p* = 0.415. Relative to Word 1 in the grammatical condition, RTs did not differ significantly at Word 2, *p* = 0.449. RTs were shorter at Word 3 than at Word 1, *t* = −2.081, *p* = 0.038. Neither the Grammaticality × Word 2 interaction (*p* = 0.528) nor the Grammaticality × Word 3 interaction (*p* = 0.299) was significant. Model-based comparisons further showed no significant grammaticality differences at Word 1, Word 2, or Word 3, *p* ≥ 0.415.

### 3.2. Results of Statistical Learning, Working Memory and Attention

Table 4 presents the descriptive results for VSL, ASL, WM, and attention. Adolescent participants performed significantly above the chance level of 0.50 in both the VSL and ASL accuracy measures, as confirmed by one-sample *t*-tests: *t*(71) = 6.86, *p* < 0.001 for VSL, and *t*(67) = 5.22, *p* < 0.001 for ASL. This indicates that participants showed learning effects in both SL tasks. In addition, VSL accuracy was significantly higher than ASL accuracy, *t*(65) = 2.17, *p* = 0.034, 95% CI for the mean difference [0.004, 0.096]. The mean VSL RT slope was negative (M = −0.0095), whereas the mean ASL RT slope was close to zero (M = 0.0000). These descriptive slope values were therefore not treated as independent evidence of learning.

For WM, participants’ OSPAN scores were calculated, with a mean score of 45.11 out of a total possible score of 75. For attention, the mean network scores were 41.59 ms for alerting, 48.16 ms for orienting, and 80.56 ms for conflict control.

### 3.3. Relationship Between Statistical Learning and L2 Morphosyntax Processing

#### 3.3.1. Correlation Analysis

The general hypotheses that SL would be associated with L2 morphosyntactic sensitivity and that these associations might vary across modalities were derived from the theoretical framework and previous research. However, no directional predictions were specified for the precise word positions or modality at which such associations would emerge. Correlation analyses were used to describe the pairwise association patterns. The six GSI measures (Word 1–Word 3 in SPR and SPL) were entered separately as depedent variables in hierarchical regression models with the same model structure.

**Figure 1:**
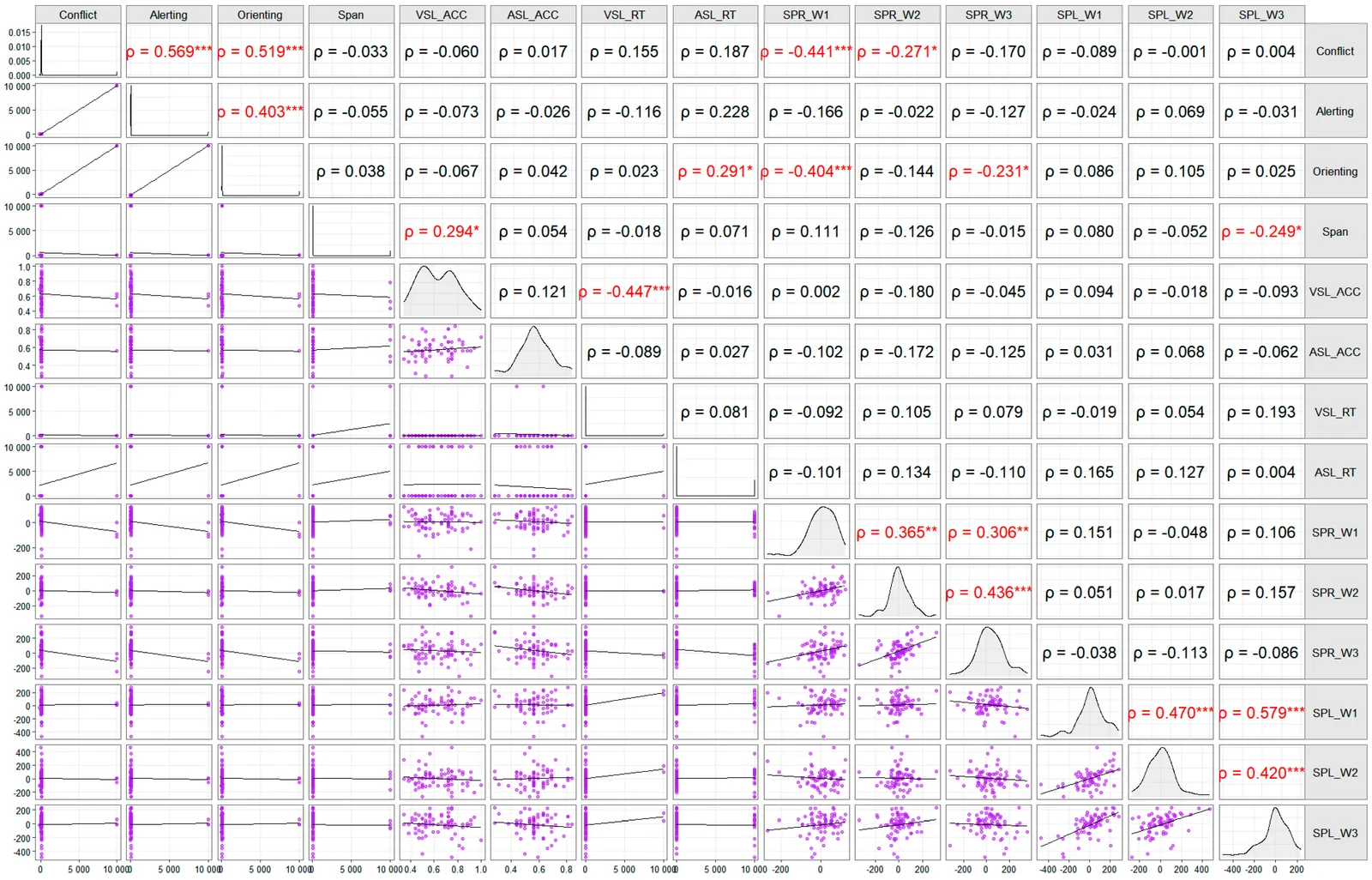
Correlation matrix showing associations between cognitive measures and L2 morphosyntactic sensitivity in the visual and auditory modalities. Values represent Spearman’s rank correlation coefficients (ρ). Correlations were calculated using pairwise complete observations; therefore, the sample size varied across variable pairs (N = 54–74). Scatterplots show pairwise relationships, and density curves on the diagonal show the distributions of individual variables. Significance markers are based on unadjusted two-tailed *p* values: * *p* < 0.05, ** *p* < 0.01, and *** *p* < 0.001.

Figure 1 presents the correlation matrix for all variables. To address multiple testing, the Benjamini–Hochberg (BH) false discovery rate correction was applied to the 12 theoretically relevant correlations between the two SL accuracy measures and the six word-position-specific GSI outcomes. VSL accuracy was positively correlated with SPR Word 3 GSI in the spillover region, *ρ* = 0.420, *p* < 0.001, BH-adjusted *p* = 0.004. No significant correlations were found between VSL accuracy and the SPL GSI measures. ASL accuracy was not significantly associated with any GSI outcome; its largest association was with SPL Word 3 GSI, *ρ* = −0.221, *p* = 0.070, BH-adjusted *p* = 0.422.

Conflict control was negatively correlated with SPL Word 1 GSI, *ρ* = −0.293, unadjusted *p* = 0.013. No other consistent associations were observed between the cognitive measures and GSI across word positions and tasks.

#### 3.3.2. Regression Predicting SPR Word 3 GSI

The same hierarchical regression procedure was applied to the Word 1, Word 2, and Word 3 GSI outcomes as dependent variables in both the SPR and SPL tasks. The predictors were entered hierarchically as follows: English midterm score and OSPAN were entered in Steps 1 and 2, respectively, followed by conflict control and orienting in Step 3. VSL and ASL accuracy were entered together in Step 4 to examine their incremental contribution after accounting for the covariates. Across the final models, tolerance values ranged from 0.733 to 0.950 and VIF values ranged from 1.053 to 1.365, indicating no problematic multicollinearity. Because the precise word position at which an SL–GSI association would emerge was not specified a priori, all word-position-specific findings were interpreted as exploratory.

To account for multiple testing, Holm correction was applied separately to two families of tests. The first family comprised the six Step 4 SL-block tests, one for each GSI outcome. The second comprised the 12 individual SL coefficients from the final models, consisting of VSL and ASL accuracy for each of the six outcomes. Because the clearest converging evidence was observed for SPR Word 3, this model is presented in the main text. Complete results for the remaining five models are reported in Supplementary File S6.

*Regression on Self-Paced Reading’s GSI at Word 3*: The hierarchical regression for SPR Word 3 included 60 participants with complete data for the outcome and all predictors entered into the model. As shown in Table 5, the final model was significant, *F*(6, 53) = 2.529, *p* = 0.032, and explained 22.3% of the variance in GSI, *R*^2^ = 0.223, adjusted *R*^2^ = 0.135. After controlling for midterm English score, span score, conflict control, and orienting, adding VSL and ASL accuracy in Step 4 accounted for an additional 15.5% of the variance, Δ*R*^2^ = 0.155, Δ*F*(2, 53) = 5.293, unadjusted *p* = 0.008. This block-level increment remained significant after Holm correction across the six SL-block tests, adjusted *p* = 0.048.

In the final model in Table 6, VSL accuracy was a significant positive predictor of SPR Word 3 GSI, *B* = 490.090, *β* = 0.442, *t*(53) = 3.223, unadjusted *p* = 0.002, 95% CI [185.133, 795.047]. This coefficient remained significant after Holm correction across the 12 individual SL coefficients, adjusted *p* = 0.026. By contrast, ASL accuracy was not a significant predictor, *B* = 66.411, *β* = 0.043, *t*(53) = 0.343, unadjusted *p* = 0.733, 95% CI [−321.502, 454.325], Holm-adjusted *p* = 1.000. Thus, the visual SL measure uniquely accounted for individual differences in morphosyntactic sensitivity in the SPR spillover region, although this word-position-specific result should be interpreted as exploratory.

A sensitivity analysis recalculated the SPR Word 3 GSI using a stricter 33-item set. Adding the two SL measures did not significantly improve the model, Δ*R*^2^ = 0.078, Δ*F*(2, 53) = 2.432, unadjusted *p* = 0.097, Holm-adjusted *p* = 0.485. The VSL coefficient was also nonsignificant, *B* = 279.316, *β* = 0.256, 95% CI [−5.728, 564.360], *t*(53) = 1.964, unadjusted *p* = 0.055, Holm-adjusted *p* = 0.605. Thus, the positive VSL–SPR Word 3 association observed in the primary analysis was not strengthened in the sensitivity analysis. Complete sensitivity regression results are reported in Supplementary File S4.

## 4. Discussion

### 4.1. Statistical Learning Performance Across Visual and Auditory Tasks

Under the fixed task sequence used in the present study, adolescent learners performed better on the VSL task than on the ASL task. This result contrasts with some previous findings based on English-speaking samples. For example, Qi et al. (2019) examined 36 typically developing children and 36 healthy adults using a classic triplet-learning paradigm in both visual and auditory modalities. Their results showed an auditory advantage in SL performance, with participants performing better in ASL than in VSL. This contrast suggests that modality preference in SL may not be universal across learner populations. Lukics and Lukács (2022) systematically compared four types of SL tasks: auditory linguistic, auditory nonlinguistic, visual linguistic, and visual nonlinguistic SL. Their results showed that when the input was presented sequentially, Hungarian-speaking adults learned the artificial grammar more successfully in the auditory than in the visual modality, with the auditory-linguistic condition showing a particularly strong advantage over the visual-linguistic condition. Hu et al. (2026) further found that English-speaking adults showed stronger ASL than VSL performance even in delayed testing, suggesting that the auditory modality may be more beneficial for SL consolidation in English speakers.

One possible explanation for the present pattern involves learners’ prior language and literacy experience. Previous studies have shown that Chinese children can extract regularities from orthographic input and that VSL is associated with Chinese orthographic awareness (He & Tong, 2017; Tong et al., 2019). Ren and Wang (2023) also reported stronger performance in some visual than auditory SL conditions among Chinese-speaking children learning English. This difference was observed in both linguistic and nonlinguistic SL tasks. It was interpreted in relation to long-term experience with visually oriented literacy practices and classroom instruction emphasizing written input. These findings raise the possibility that experience with Chinese orthography and visually oriented classroom instruction may be related to visual SL performance. However, because the task order was fixed, the observed VSL–ASL difference cannot be attributed uniquely to modality. Moreover, the present study did not directly measure participants’ orthographic-processing preferences or their relative exposure to visual and auditory English input. These factors therefore remain tentative explanations.

### 4.2. L2 Morphosyntax Processing in Visual and Auditory Tasks

The task-specific analyses provided no statistically reliable evidence of grammaticality-related processing in either SPR or SPL. In SPR, the Grammaticality × Word Position interaction at Word 3 was not significant. Descriptively, however, RTs at Word 3 were longer for ungrammatical than for grammatical sentences. In SPL, no grammaticality-related effect was observed at any word position.

The location of this descriptive SPR pattern is compatible with previous accounts of spillover processing. Processing difficulty triggered at a critical morphosyntactic form may not become observable until subsequent words (Marsden et al., 2018). H. Kim et al. (2023) similarly found that child L2 learners showed sensitivity to mismatching Korean numeral-quantifier constructions in spillover rather than critical regions. However, the nonsignificant interaction in the present study prevents the Word 3 pattern from being interpreted as reliable evidence of spillover processing. Differences in learner population, target language, linguistic structure, and learning context limit direct comparison between the two studies. The present findings therefore suggest only the possibility of delayed sensitivity to L2 inflectional violations in reading, which requires replication with stronger statistical evidence.

The absence of a reliable SPL effect differs from Marinis (2007), who found that Turkish-speaking children learning English as an additional language used morphological cues while processing English active and passive sentences in a picture-supported SPL task. That task provided a visual event context, whereas the present task required learners to process low-salience auditory inflections without concurrent pictorial support. Differences in L1 morphology and learning experience may also have contributed, but these factors were not directly tested.

To date, there appears to be little direct evidence comparing adolescent L2 learners’ implicit morphosyntax processing across reading and listening input. Listening input is temporally transient and externally paced, which makes it more difficult for L2 learners to allocate attention simultaneously to meaning and low-salience grammatical forms. This difficulty may be particularly pronounced for Chinese EFL adolescents, whose English learning experience is mainly classroom-based and visually oriented. Ren and Wang (2023), whose sample was highly relevant to the present study, found that Chinese-speaking children learning English as an L2 performed better in English VSL than in English ASL. Their teacher questionnaire also showed that English instruction emphasized reading, writing, and vocabulary more than speaking and listening. However, the relative amount of visual and auditory instruction received by the present participants was not measured. Classroom experience therefore remains a tentative explanation. Moreover, because the difference between the SPR and SPL patterns was not directly tested, the present results should not be interpreted as evidence of a modality difference.

### 4.3. Associations Between Statistical Learning and L2 Morphosyntax Processing

The primary regression analyses identified one statistically significant but exploratory association. For SPR Word 3, the final model explained 22.3% of the variance in GSI, with an adjusted *R*^2^ of 13.5%. Adding the two SL measures accounted for an additional 15.5% of the variance, and VSL accuracy was positively associated with SPR Word 3 GSI, *β* = 0.442. None of the SL blocks or individual SL coefficients in the other five word-position-specific models remained significant after correction. The positive VSL–SPR Word 3 association was not strengthened in the stricter sensitivity analysis. The primary finding should therefore be interpreted cautiously.

Previous studies with L1 children have shown that individual differences in SL are associated with syntactic acquisition, syntactic comprehension, and broader language development (Donnelly et al., 2024; Garcia et al., 2021; Kidd, 2012; Kidd et al., 2023; Kidd & Arciuli, 2016; Kumarage et al., 2022). Kidd and Arciuli (2016) used a nonlinguistic VSL task and found that children’s SL performance predicted their comprehension of syntactically demanding structures, particularly passives and object relative clauses. This finding suggests that children who are more sensitive to distributional regularities may be better able to acquire and process low-frequency or structurally complex syntactic patterns.

Direct evidence on SL as a mechanism supporting child or adolescent L2 morphosyntax processing remains limited. Pili-Moss (2021) examined 8- to 9-year-old children in the earliest stages of exposure to a novel L2. The study found that the serial reaction time task—a measure of implicit learning ability—predicted children’s learning of L2 word order and increasingly supported aural sentence comprehension as practice progressed. More directly related to adolescent classroom learning, Pili-Moss et al. (2025) found that VSL, in interaction with WM, predicted EFL direct-question development and written-task outcomes in 12- to 13-year-old secondary school learners. Although these studies did not examine online morphosyntactic violation sensitivity in natural L2 sentence processing, they provide relevant evidence that implicit learning mechanisms can contribute to L2 grammar development in younger learners.

The positive association between VSL accuracy and SPR Word 3 GSI is consistent with the possibility that sensitivity to visual regularities is related to individual differences in processing visually presented L2 input. However, because the regression design was correlational and the associations across the visual and auditory tasks were not compared directly, the present findings do not establish modality specificity. Replication using preregistered word regions, counterbalanced task orders, and larger samples is needed.

### 4.4. Limited Contribution of Working Memory and Attention

WM and attention did not make consistent independent contributions to L2 morphosyntactic sensitivity across tasks or word positions. This pattern is compatible with evidence that the contribution of WM and executive functions to L2 learning may depend on learners’ age, task demands, and their interactions with other learning abilities rather than emerge as stable independent effects (Kapa & Colombo, 2014; Pili-Moss et al., 2025; Stone & Pili-Moss, 2016). The present findings should therefore be interpreted as an absence of reliable evidence in these tasks, rather than evidence that WM and attention play no role in L2 processing.

## 5. Conclusions

This study examined whether SL explains individual differences in L2 morphosyntax processing among Chinese adolescent EFL learners after accounting for WM and attention. The mixed-effects analyses provided no statistically reliable evidence of grammaticality-related processing in either SPR or SPL, although SPR showed a descriptive pattern of longer RTs for ungrammatical sentences at Word 3. In the exploratory regression analyses, VSL accuracy positively predicted SPR Word 3 GSI in the primary analysis, but it was not strengthened in the sensitivity analysis. No other SL predictor showed a reliable association with the GSI outcomes, and WM and attention did not make consistent independent contributions. Overall, the findings provide preliminary evidence that SL may contribute to individual differences in adolescent L2 morphosyntax processing. The study extends individual-differences research to younger, classroom-based L2 learners using online sentence-processing measures.

Several limitations should be acknowledged. First, the cross-sectional design does not permit causal inference. Moreover, the a priori power analysis was based on a bivariate correlation rather than the six-predictor hierarchical regression, for which only 60 complete cases were available. The regression findings should therefore require replication in a larger sample prospectively powered for the full model. Second, participants were drawn from three classes at one junior secondary school in South China, limiting the generalizability of the findings. Dialect experience, orthographic-processing preferences, and relative exposure to visual and auditory English input were not measured directly, so explanations based on these factors remain tentative. Third, the visual and auditory tasks were administered in a fixed order and differed in their stimuli, exposure schedules, and measurement reliability. Consequently, differences between VSL and ASL or between SPR and SPL cannot be attributed uniquely to modality. Future research should use counterbalanced task orders and more closely matched visual and auditory measures. Finally, although invalid items were removed following a post hoc linguistic audit, the materials were not independently normed for predictability and plausibility before data collection. The quality of some comprehension questions may also have influenced comprehension accuracy and the associated trial exclusions. Future studies should prospectively validate both the critical sentences and comprehension questions before administration.

## Figures and Tables

**Table 1:** The three grammatical structures and example sentences.

Grammar Structure	Example of Grammatical Sentence	Example of Ungrammatical Sentence	Pedagogical Introduction
Third-person singular agreement	She always * remembers to check her email before leaving.	She always * remember to check her email before leaving.	Elementary/ Lower intermediate
Plural marking	The fire damaged a number of the * books in the library.	The fire damaged a number of the * book in the library.	No clear focus at any level
Past-tense marking	Yesterday, I * helped a lost child find his way.	Yesterday, I * help a lost child find his way.	Elementary/ Lower intermediate

* represents the position where grammatical violation may appear.

**Table 2:** Linear mixed-effects model for SPR.

Effect	*β*	SE	*t*	*p*
Intercept	6.310	0.037	168.477	<0.001
Grammaticality	0.008	0.016	0.476	0.635
Word 2 vs. Word 1	0.029	0.014	1.978	0.050
Word 3 vs. Word 1	0.083	0.019	4.398	<0.001
Grammaticality × Word 2	0.001	0.018	0.082	0.935
Grammaticality × Word 3	0.032	0.018	1.729	0.084

**Table 3:** Linear mixed-effects model for SPL.

Effect	*β*	SE	*t*	*p*
Intercept	6.877	0.047	144.980	<0.001
Grammaticality	0.018	0.023	0.817	0.415
Word 2 vs. Word 1	−0.012	0.016	−0.758	0.449
Word 3 vs. Word 1	−0.034	0.016	−2.081	0.038
Grammaticality × Word 2	−0.015	0.023	−0.630	0.528
Grammaticality × Word 3	−0.024	0.023	−1.039	0.299

**Table 4:** The descriptive statistics of the cognitive measures.

Measures	Mean Value	SD	Valid N
VSL accuracy	0.63	0.16	72
VSL RT slope	−0.0095	0.0482	72
ASL accuracy	0.57	0.12	68
ASL RT slope	0.0000	0.0223	56 *
Working Memory	45.11 (Span score)	14.55	70
Alerting	41.59 (RT difference)	18.06	71
Orienting	48.16 (RT difference)	22.88	71
Conflict control	80.56 (RT difference)	35.40	71

*Note.* Valid N refers to the number of participants contributing usable data to each measure. * RT slope scores were calculated only for participants with at least 60% valid target-detection trials. A participant’s RT-slope measure was treated as missing when more than 40% of the target-detection trials were invalid. Responses occurring outside the prespecified temporal window extending 480 ms before and 480 ms after the target stimulus were coded as invalid. This criterion resulted in a substantially smaller valid sample for the ASL RT slope measure, reflecting the relatively high frequency of responses falling outside the auditory response window. Participants whose RTslope scores were unavailable remained eligible for analyses involving their other valid measures. All other task-level missing data resulted from equipment-related data loss during school-based testing, despite equipment checks conducted before each experimental session.

**Table 5:** Model summary for predicting GSI in visual SPR spillover region at Word 3.

Step	Predictors Entered	*R* ^2^	Adjusted *R*^2^	Δ*R*^2^	Δ*F*	*p* for Δ*F*	Model *F*	Model *p*
1	Midterm score	0.018	0.001	0.018	1.05	0.31	1.05	0.31
2	Span score	0.058	0.025	0.04	2.449	0.123	1.763	0.181
3	Conflict control, Orienting	0.067	−0.001	0.009	0.266	0.768	0.991	0.42
4	ASL accuracy, VSL accuracy	0.223	0.135	0.155	5.293	0.008	2.529	0.032

*Note.* The dependent variable was GSI at Word 3 in the visual SPR task.

**Table 6:** Final-model coefficients for GSI in SPR at Word 3.

Predictor	*B*	SE	*β*	95% CI for *B*	*t*	Unadjusted *p*	Holm-Adjusted *p*	VIF	Tolerance
Midterm English score	0.375	2.947	0.016	[−5.536, 6.286]	0.127	0.899	—	1.127	0.887
Span score	0.460	1.652	0.037	[−2.854, 3.775]	0.278	0.782	—	1.219	0.820
Conflict control	0.391	0.665	0.082	[−0.943, 1.726]	0.588	0.559	—	1.323	0.756
Orienting	0.145	1.052	0.019	[−1.966, 2.255]	0.137	0.891	—	1.365	0.733
VSL accuracy	490.090	152.042	0.442	[185.133, 795.047]	3.223	0.002	0.026	1.282	0.780
ASL accuracy	66.411	193.401	0.043	[−321.502, 454.325]	0.343	0.733	1	1.056	0.947

*Note.* CI = confidence interval, VIF = variance inflation factor.

## Data Availability

The data are available from the corresponding author upon reasonable request because the dataset includes academic performance scores from adolescent participants, which are subject to school confidentiality policies and parental informed consent conditions. De-identified data may be shared for legitimate academic purposes.

## Supplementary Materials

The following supporting information can be downloaded at: https://www.mdpi.com/article/10.3390/bs16081452/s1, Supplementary File S1: Previous Studies of Statistical Learning and L2 Morphosyntax; Supplementary File S2: Participant Demographic and Language-Background Characteristics; Supplementary File S3: Original Experimental Materials and Post Hoc Linguistic Evaluation; Supplementary File S4: Sensitivity Analyses of the SPR and SPL Mixed-Effects Models and Hierarchical Regressions; Supplementary File S5: R Code and Random-Effects Simplification for the SPR and SPL Models; Supplementary File S6: Complete Results of the Five Primary Hierarchical Regression Analyses.

## Abbreviations

The following abbreviations are used in this manuscript:

**Abbreviation**	**Definition**
2AFC	Two-alternative forced-choice task
ACC	Accuracy
ASL	Auditory statistical learning
BH	Benjamini–Hochberg
CI	Confidence interval
EFL	English as a foreign language
GSI	Grammatical Sensitivity Index
L1	First language
L2	Second language
OSPAN	Operation span task
RT	Reaction time
SL	Statistical learning
SPL	Self-paced listening
SPR	Self-paced reading
VIF	Variance inflation factor
VSL	Visual statistical learning
WM	Working memory

## References

[B1-behavsci-16-01452] Chen Y., Li L., Wang M., Wang R. (2023). The association between statistical learning and the development of second language grammar learning. Applied Cognitive Psychology.

[B2-behavsci-16-01452] Chen Y., Li L., Yang J., Wang M., Wang R. (2026). The neural mechanism underlying statistical learning’s contribution to L2 grammar learning. Brain and Language.

[B3-behavsci-16-01452] Conway C. M., Christiansen M. H. (2005). Modality-constrained statistical learning of tactile, visual, and auditory sequences. Journal of Experimental Psychology: Learning, Memory, and Cognition.

[B4-behavsci-16-01452] Coughlin C. E., Tremblay A. (2013). Proficiency- and working-memory-based explanations for nonnative speakers’ sensitivity to agreement in sentence processing. Applied Psycholinguistics.

[B5-behavsci-16-01452] Coumel M., Ushioda E., Messenger K. (2023). Second language learning via syntactic priming: Investigating the role of modality, attention, and motivation. Language Learning.

[B6-behavsci-16-01452] Donnelly S., Rowland C., Chang F., Kidd E. (2024). A comprehensive examination of prediction-based error as a mechanism for syntactic development: Evidence from syntactic priming. Cognitive Science.

[B7-behavsci-16-01452] Ellis N. C. (2008). The dynamics of second language emergence: Cycles of language use, language change, and language acquisition. The Modern Language Journal.

[B8-behavsci-16-01452] Ettlinger M., Morgan-Short K., Faretta-Stutenberg M., Wong P. C. M. (2016). The relationship between artificial and second language learning. Cognitive Science.

[B9-behavsci-16-01452] Fan J., McCandliss B. D., Sommer T., Raz A., Posner M. I. (2002). Testing the efficiency and independence of attentional networks. Journal of Cognitive Neuroscience.

[B10-behavsci-16-01452] Faretta-Stutenberg M., Morgan-Short K. (2018). The interplay of individual differences and context of learning in behavioral and neurocognitive second language development. Second Language Research.

[B11-behavsci-16-01452] Garcia R., Garrido Rodriguez G., Kidd E. (2021). Developmental effects in the online use of morphosyntactic cues in sentence processing: Evidence from Tagalog. Cognition.

[B12-behavsci-16-01452] Godfroid A., Kim K. M. (2021). The contributions of implicit-statistical learning aptitude to implicit second-language knowledge. Studies in Second Language Acquisition.

[B13-behavsci-16-01452] Granena G. (2013). Individual differences in sequence learning ability and second language acquisition in early childhood and adulthood. Language Learning.

[B14-behavsci-16-01452] He X., Tong X. (2017). Statistical learning as a key to cracking Chinese orthographic codes. Scientific Studies of Reading.

[B15-behavsci-16-01452] Hu A., Parks K. M. A., Schulz S. E., Larmer S. M. H., Batterink L. J., Stevenson R. A. (2026). Modality-specific consolidation shapes long-term retention in statistical learning. Psychological Research.

[B16-behavsci-16-01452] Jegerski J., Jegerski J., VanPatten B. (2013). Self-paced reading. Research methods in second language psycholinguistics.

[B17-behavsci-16-01452] Jiang N. (2004). Morphological insensitivity in second language processing. Applied Psycholinguistics.

[B18-behavsci-16-01452] Jiang N. (2007). Selective integration of linguistic knowledge in adult second language learning. Language Learning.

[B19-behavsci-16-01452] Just M. A., Carpenter P. A. (1992). A capacity theory of comprehension: Individual differences in working memory. Psychological Review.

[B20-behavsci-16-01452] Kapa L. L., Colombo J. (2014). Executive function predicts artificial language learning. Journal of Memory and Language.

[B21-behavsci-16-01452] Keating G. D. (2009). Sensitivity to violations of gender agreement in native and nonnative Spanish: An eye-movement investigation. Language Learning.

[B22-behavsci-16-01452] Kidd E. (2012). Implicit statistical learning is directly associated with the acquisition of syntax. Developmental Psychology.

[B23-behavsci-16-01452] Kidd E., Arciuli J. (2016). Individual differences in statistical learning predict children’s comprehension of syntax. Child Development.

[B24-behavsci-16-01452] Kidd E., Arciuli J., Christiansen M. H., Smithson M. (2023). The sources and consequences of individual differences in statistical learning for language development. Cognitive Development.

[B25-behavsci-16-01452] Kim H., Kim K., Jo K., Hwang H. (2023). Sensitivity to syntactic dependency formation in child second language processing: A study of numeral quantifiers in Korean. Language and Cognition.

[B26-behavsci-16-01452] Kim J., Cho Y., Cho Y., Hong Y., Kim S., Nam H. (2020). The effects of L1–L2 phonological mappings on L2 phonological sensitivity: Evidence from self-paced listening. Studies in Second Language Acquisition.

[B27-behavsci-16-01452] Kim K. M., Godfroid A. (2019). Should we listen or read? Modality effects in implicit and explicit knowledge. The Modern Language Journal.

[B28-behavsci-16-01452] Kumarage S., Donnelly S., Kidd E. (2022). Implicit learning of structure across time: A longitudinal investigation of syntactic priming in young English-acquiring children. Journal of Memory and Language.

[B29-behavsci-16-01452] Lee O.-S. (2023). Implicit statistical learning in L2 sentence processing: Individual cognitive differences. Journal of Psycholinguistic Research.

[B30-behavsci-16-01452] Lukács Á., Dobó D., Szőllősi Á., Németh K., Lukics K. S. (2023). Reading fluency and statistical learning across modalities and domains: Online and offline measures. PLoS ONE.

[B31-behavsci-16-01452] Lukics K. S., Lukács Á. (2022). Modality, presentation, domain and training effects in statistical learning. Scientific Reports.

[B32-behavsci-16-01452] Marinis T., Belikova A., Meroni L., Umeda M. (2007). On-line processing of passives in L1 and L2 children. Proceedings of the 2nd conference on generative approaches to language acquisition North America (GALANA).

[B33-behavsci-16-01452] Marsden E., Thompson S., Plonsky L. (2018). A methodological synthesis of self-paced reading in second language research. Applied Psycholinguistics.

[B34-behavsci-16-01452] McDonald J. L. (2006). Beyond the critical period: Processing-based explanations for poor grammaticality judgment performance by late second language learners. Journal of Memory and Language.

[B35-behavsci-16-01452] McDonough K., Trofimovich P. (2016). The role of statistical learning and working memory in L2 speakers’ pattern learning. The Modern Language Journal.

[B36-behavsci-16-01452] Meulman N., Stowe L. A., Sprenger S. A., Bresser M., Schmid M. S. (2014). An ERP study on L2 syntax processing: When do learners fail?. Frontiers in Psychology.

[B37-behavsci-16-01452] Misyak J. (2010). On-line individual differences in statistical learning predict language processing. Frontiers in Psychology.

[B38-behavsci-16-01452] Misyak J., Christiansen M. H. (2012). Statistical learning and language: An individual differences study. Language Learning.

[B39-behavsci-16-01452] Novick J. M., Trueswell J. C., Thompson-Schill S. L. (2005). Cognitive control and parsing: Reexamining the role of Broca’s area in sentence comprehension. Cognitive, Affective, & Behavioral Neuroscience.

[B40-behavsci-16-01452] Novick J. M., Trueswell J. C., Thompson-Schill S. L. (2010). Broca’s area and language processing: Evidence for the cognitive control connection. Language and Linguistics Compass.

[B41-behavsci-16-01452] Papadopoulou D., Tsimpli I., Amvrazis N., Jegerski J., VanPatten B. (2013). Self-paced listening. Research methods in second language psycholinguistics.

[B42-behavsci-16-01452] Pili-Moss D. (2021). Cognitive predictors of child second language comprehension and syntactic learning. Language Learning.

[B43-behavsci-16-01452] Pili-Moss D., Hamrick P., Wendebourg K., Schmidt T., Meurers D. (2025). Implicit statistical learning and working memory predict EFL development and written task outcomes in adolescents. System.

[B44-behavsci-16-01452] Qi Z., Sanchez Araujo Y., Georgan W. C., Gabrieli J. D. E., Arciuli J. (2019). Hearing matters more than seeing: A cross-modality study of statistical learning and reading ability. Scientific Studies of Reading.

[B45-behavsci-16-01452] Ren J., Wang M. (2023). Development of statistical learning ability across modalities, domains, and languages. Journal of Experimental Child Psychology.

[B46-behavsci-16-01452] Ren J., Wang M., Arciuli J. (2023). A meta-analysis on the correlations between statistical learning, language, and reading outcomes. Developmental Psychology.

[B47-behavsci-16-01452] Schmidt R. (2012). Attention, awareness, and individual differences in language learning. Perspectives on Individual Characteristics and Foreign Language Education.

[B48-behavsci-16-01452] Schneider J. M., Hu A., Legault J., Qi Z. (2020). Measuring statistical learning across modalities and domains in school-aged children via an online platform and neuroimaging techniques. Journal of Visualized Experiments.

[B49-behavsci-16-01452] Siegelman N., Bogaerts L., Elazar A., Arciuli J., Frost R. (2018). Linguistic entrenchment: Prior knowledge impacts statistical learning performance. Cognition.

[B50-behavsci-16-01452] Song Y. (2015). L2 processing of plural inflection in English. Language Learning.

[B51-behavsci-16-01452] Spencer M., Kaschak M. P., Jones J. L., Lonigan C. J. (2015). Statistical learning is related to early literacy-related skills. Reading and Writing.

[B52-behavsci-16-01452] Stone H., Pili-Moss D. (2016). Executive function and language learning: Differentiating vocabulary and morpho-syntax. Lancaster Postgraduate Conference in Linguistics and Language Teaching.

[B53-behavsci-16-01452] Suzuki Y., DeKeyser R. (2017). The interface of explicit and implicit knowledge in a second language: Insights from individual differences in cognitive aptitudes. Language Learning.

[B54-behavsci-16-01452] Suzuki Y., Jeong H., Cui H., Okamoto K., Kawashima R., Sugiura M. (2023). An fMRI validation study of the word-monitoring task as a measure of implicit knowledge: Exploring the role of explicit and implicit aptitudes in behavioral and neural processing. Studies in Second Language Acquisition.

[B55-behavsci-16-01452] Suzuki Y., Sunada M. (2018). Automatization in second language sentence processing: Relationship between elicited imitation and maze tasks. Bilingualism: Language and Cognition.

[B56-behavsci-16-01452] Tong X., Leung W. W. S., Tong X. (2019). Visual statistical learning and orthographic awareness in Chinese children with and without developmental dyslexia. Research in Developmental Disabilities.

[B57-behavsci-16-01452] Unsworth N., Heitz R. P., Schrock J. C., Engle R. W. (2005). An automated version of the operation span task. Behavior Research Methods.

[B58-behavsci-16-01452] Veenstra A., Antoniou K., Katsos N., Kissine M. (2018). Resisting attraction: Individual differences in executive control are associated with subject–verb agreement errors in production. Journal of Experimental Psychology: Learning, Memory, and Cognition.

[B59-behavsci-16-01452] White L. (2003). Fossilization in steady state L2 grammars: Persistent problems with inflectional morphology. Bilingualism: Language and Cognition.

[B60-behavsci-16-01452] Ye Z., Zhou X. (2009). Executive control in language processing. Neuroscience & Biobehavioral Reviews.

[B61-behavsci-16-01452] Zalbidea J. (2021). On the scope of output in SLA: Task modality, salience, L2 grammar noticing, and development. Studies in Second Language Acquisition.

[B62-behavsci-16-01452] Zalbidea J., Sanz C. (2020). Does learner cognition count on modality? Working memory and L2 morphosyntactic achievement across oral and written tasks. Applied Psycholinguistics.

[B63-behavsci-16-01452] Zhao C., Kormos J., Rebuschat P., Suzuki S. (2021). The role of modality and awareness in language learning. Applied Psycholinguistics.

[B64-behavsci-16-01452] Zhuang B., Hu S., Liang L. (2025). Attention to L2 morpho-syntactic form across visual and aural modalities and the modulation of working memory: ERP evidence. Journal of Neurolinguistics.

