# Supplementary material for: The Contributions of Statistical Learning to L2 Morphosyntax Processing Across Visual and Auditory Modalities in Chinese Adolescent Learners
Source: Behav Sci (Basel). 2026 Aug 21;16(8):1452. doi: 10.3390/bs16081452 (PMC13509579; doi:10.3390/bs16081452)
Supplement: Supplementary file 1 [file behavsci-16-01452-s001.zip › Supplementary File S4_Sensitivity Analyses of the SPR and SPL Mixed-Effects Models and Hierarchical Regressions.pdf]

Supplementary File S4. Sensitivity Analyses of the SPR and SPL Mixed-Effects Models and Hierarchical Regressions

S1. Sensitivity Analyses of the Linear Mixed-Effects Models

The primary analyses excluded five items judged to contain linguistic problems that could invalidate the intended grammaticality contrast. To assess the robustness of the findings, the sensitivity analyses additionally excluded 10 items flagged for less severe sentence-level or comprehension-question concerns. Thus, the sensitivity datasets retained 33 of the original 48 target items. The item-level audit decisions and reasons for exclusion are reported in Supplementary File S3.

Table S3. Item and observation exclusions in the primary and sensitivity analyses

Task	Analysis version	Items excluded	Sentence trials excluded	Word-region observations excluded
SPR	Primary analysis	5	283	805
SPR	Sensitivity analysis: additional	10	497	1423
SPR	Sensitivity analysis: cumulative	15	780	2228
SPL	Primary analysis	5	264	768
SPL	Sensitivity analysis: additional	10	493	1432
SPL	Sensitivity analysis: cumulative	15	757	2200

Note. Cumulative sensitivity-analysis exclusions comprise the five items removed from the primary analysis and the 10 additionally flagged items.

S1.1. SPR Sensitivity Analysis

The SPR sensitivity analysis included 76 participants, 33 items, and 6,472 word-region observations. Word-level RTs were natural-log transformed. The model included grammaticality, word position, and their interaction as fixed effects, with grammatical sentences and Word 1 serving as the reference levels. The random-effects structure was the same as in the primary SPR model, with participant-level slopes for grammaticality and Word 3 and item-level slopes for grammaticality, Word 2, and Word 3. The model converged and was not singular.

As shown in Table 2, the main effect of grammaticality at Word 1 was not significant, $\beta = 0.013$, $SE = 0.018$, $t = 0.70$, $p = .484$. RTs at Word 2 did not differ significantly from those at Word 1, $\beta = 0.030$, $SE = 0.017$, $t = 1.83$, $p = .070$, whereas RTs at Word 3 were significantly longer than those at Word 1, $\beta = 0.116$, $SE = 0.019$, $t = 6.16$, $p < .001$.

Neither the Grammaticality × Word 2 interaction, $\beta = 0.004$, $SE = 0.020$, $t = 0.20$, $p = .842$, nor the Grammaticality × Word 3 interaction, $\beta = 0.029$, $SE = 0.020$, $t = 1.41$, $p = .158$, was significant. A model-based comparison showed longer RTs for ungrammatical than grammatical sentences at Word 3, $\beta = 0.042$, $SE = 0.018$, $t = 2.31$, $p = .022$.

Table S4. Linear mixed-effects model for SPR: sensitivity analysis.

Effect	*β*	SE	*t*	*p*
Intercept	6.321	0.039	161.786	< .001
Grammaticality	0.013	0.018	0.701	.484
Word 2 vs. Word 1	0.030	0.017	1.831	.070
Word 3 vs. Word 1	0.116	0.019	6.155	< .001
Grammaticality × Word 2	0.004	0.020	0.200	.842
Grammaticality × Word 3	0.029	0.020	1.413	.158

Note. The analysis included 6472 word-region observations from 76 participants and 33 items.Grammatical sentences and Word 1 were the reference levels. RTs were natural-log transformed.

S1.2 . SPL Sensitivity Analysis

The SPL sensitivity analysis included 70 participants, 33 items, and 6,005 word-region observations. Word-level RTs were natural-log transformed. The fixed-effects specification and reference levels were the same as in the SPR analysis. The random-effects structure retained grammaticality slopes for both participants and items. The model converged and was not singular.

As shown in Table 3, the main effect of grammaticality at Word 1 was not significant, $\beta = 0.021$, $SE = 0.025$, $t = 0.85$, $p = .398$. RTs at Word 2 and Word 3 did not differ significantly from those at Word 1, $p = .276$ and .124, respectively. Neither the Grammaticality × Word 2 interaction, $\beta = -0.008$, $SE = 0.026$, $t = -0.30$, $p = .765$, nor the Grammaticality × Word 3 interaction, $\beta = -0.034$, $SE = 0.026$, $t = -1.31$, $p = .189$, was significant.

Model-based comparisons likewise showed no significant grammaticality difference at Word 1, Word 2, or Word 3, with $p \geq .398$. Thus, excluding the additionally flagged items did not change the SPL conclusion: there was no reliable grammaticality-related effect at any word position.

Table S5. Linear mixed-effects model for SPL: sensitivity analysis。

Effect	*β*	SE	*t*	*p*
Intercept	6.880	0.048	144.014	< .001
Grammaticality	0.021	0.025	0.848	.398
Word 2 vs. Word 1	−0.020	0.018	−1.089	.276

Word 3 vs. Word 1	−0.028	0.018	−1.540	.124
Grammaticality × Word 2	−0.008	0.026	−0.299	.765
Grammaticality × Word 3	−0.034	0.026	−1.314	.189

Note. The analysis included 6,005 word-region observations from 70 participants and 33 items. Grammatical sentences and Word 1 were the reference levels. RTs were natural-log transformed.

S2. Sensitivity Analyses of the Hierarchical Regressions

Holm correction was applied separately across the six Step 4 SL-block tests and the 12 individual VSL/ASL coefficients.

Table S6a. Model summary for predicting GSI in SPR at Word 1.

Step	Predictors entered	R^2	Adjusted R^2	ΔR^2	ΔF	Unadjusted *p* for ΔF	Holm-adjusted *p*	Model *F*	Model *p*
1	Midterm English score	.120	.105	.120	8.169	.006	—	8.169	.006
2	Span score	.147	.118	.028	1.905	.173	—	5.099	.009
3	Conflict control, orienting	.268	.216	.120	4.688	.013	—	5.212	.001
4	VSL accuracy, ASL accuracy	.284	.206	.016	.618	.543	1.000	3.634	.004

Note. N = 60; residual degree of freedom = 53 for all SPR model.

Table S6b. Final-model coefficients for GSI in SPR at Word 1.

Predictor	B	SE	*β*	95% CI for B	*t*	Unadjusted *p*	Holm-adjusted *p*	VIF	Tolerance
Midterm English score	-3.460	1.071	-.388	[-5.607, -1.314]	-3.230	.002	—	1.106	.905
Span score	1.059	0.601	.219	[-0.145, 2.264]	1.763	.084	—	1.190	.840
Conflict control	-0.304	0.246	-.161	[-0.797, 0.190]	-1.232	.223	—	1.313	.762
Orienting	-0.767	0.390	-.262	[-1.549, 0.014]	-1.968	.054	—	1.358	.736

**Predictor**	B	**SE**	***β***	**95% CI for B**	***t***	**Unadjusted *p***	**Holm-adjusted *p***	**VIF**	**Tolerance**
VSL accuracy	-49.231	72.036	-.080	[-193.594, 95.132]	-.683	.497	1.000	1.053	.950
ASL accuracy	-46.605	55.133	-.107	[-157.095, 63.884]	-.845	.402	1.000	1.223	.818

Note.CI = confidence interval; VIF = variance inflation factor.

Table S7a. Model summary for predicting GSI in SPR at Word 2.

Step	Predictors entered	R^2	Adjusted R^2	ΔR^2	ΔF	Unadjusted p for ΔF	Holm-adjusted p	Model F	Model p
1	Midterm English score	.027	.011	.027	1.652	.204	—	1.652	.204
2	Span score	.079	.048	.052	3.354	.072	—	2.535	.088
3	Conflict control, orienting	.080	.016	.001	.029	.971	—	1.241	.304
4	VSL accuracy, ASL accuracy	.163	.072	.083	2.740	.073	.438	1.791	.118

Table S7b. Final-model coefficients for GSI in SPR at Word 2.

Predictor	B	SE	β	95% CI for B	t	Unadjusted p	Holm-adjusted p	VIF	Tolerance
Midterm English score	-0.806	1.661	-.063	[-4.134, 2.522]	-.485	.629	—	1.106	.905
Span score	-1.260	0.932	-.182	[-3.128, 0.607]	-1.353	.182	—	1.190	.840
Conflict control	-0.106	0.382	-.039	[-0.871, 0.659]	-.278	.782	—	1.313	.762
Orienting	-0.058	0.605	-.014	[-1.269, 1.154]	-.096	.924	—	1.358	.736
VSL accuracy	-225.682	111.674	-.256	[-449.482, -1.882]	-2.021	.048	.576	1.053	.950
ASL accuracy	-93.061	85.471	-.149	[-264.349, 78.227]	-1.089	.281	1.000	1.223	.818

*Note.*CI = confidence interval; VIF = variance inflation factor.

Table S8a. Model summary for predicting GSI in SPR at Word 3.

Step	Predictors entered	R^2	Adjusted R^2	ΔR^2	ΔF	Unadjusted p for ΔF	Holm-adjusted p	Model F	Model p
1	Midterm English score	.012	-.004	.012	.751	.390	—	.751	.390
2	Span score	.014	-.020	.001	.070	.792	—	.405	.669
3	Conflict control, orienting	.037	-.030	.024	.705	.498	—	.553	.698
4	VSL accuracy, ASL accuracy	.116	.019	.078	2.432	.097	.485	1.198	.321

Table S8b. Final-model coefficients for GSI in SPR at Word 3.

Predictor	B	SE	β	95% CI for B	t	Unadjusted p	Holm-adjusted p	VIF	Tolerance
Midterm English score	-1.184	2.115	-.075	[-5.422, 3.055]	-.560	.578	—	1.106	.905
Span score	0.826	1.187	.096	[-1.552, 3.205]	.696	.489	—	1.190	.840
Conflict control	0.230	0.486	.069	[-0.745, 1.204]	.472	.639	—	1.313	.762
Orienting	-1.128	0.770	-.217	[-2.671, 0.415]	-1.465	.149	—	1.358	.736
VSL accuracy	279.316	142.235	.256	[-5.728, 564.360,]	1.964	.055	.605	1.053	.950
ASL accuracy	-99.521	108.861	-.128	[-317.683, 118.640]	-.914	.365	1.000	1.223	.818

*Note.*CI = confidence interval; VIF = variance inflation factor.

Table S9a. Model summary for predicting GSI in SPL at Word 1.

Step	Predictors entered	R^2	Adjusted R^2	ΔR^2	ΔF	Unadjusted *p* for ΔF	Holm-adjusted *p*	Model *F*	Model *p*
1	Midterm English score	.001	-.016	.001	.052	.821	—	.052	.821
2	Span score	.011	-.023	.010	.606	.439	—	.329	.721
3	Conflict control, orienting	.024	-.044	.013	.384	.683	—	.353	.841
4	VSL accuracy, ASL accuracy	.028	-.078	.004	.105	.900	1.000	.263	.952

*Note.*N = 62; residual degree of freedom = 55 for the final SPL Word 1 model.

Table S9b. Final-model coefficients for GSI in SPL at Word 1.

Predictor	B	SE	β	95% CI for B	*t*	Unadjusted *p*	Holm-adjusted *p*	VIF	Tolerance
Midterm English score	-0.794	2.455	-.045	[-5.713, 4.126]	-.323	.748	—	1.106	.905
Span score	0.628	1.377	.066	[-2.132, 3.389]	.456	.650	—	1.190	.840
Conflict control	-0.467	0.564	-.126	[-1.599, 0.664]	-.828	.411	—	1.313	.762
Orienting	0.590	0.894	.102	[-1.201, 2.381]	.660	.512	—	1.358	.736
VSL accuracy	-21.949	165.084	-.018	[-352.786, 308.887]	-.133	.895	1.000	1.053	.950
ASL accuracy	56.159	126.349	.065	[-197.051, 309.368]	.444	.658	1.000	1.223	.818

*Note.*CI = confidence interval; VIF = variance inflation factor.

Table S10a. Model summary for predicting GSI in SPL at Word 2.

Step	Predictors entered	R^2	Adjusted R^2	ΔR^2	ΔF	Unadjusted *p* for ΔF	Holm-adjusted *p*	Model *F*	Model *p*
1	Midterm English score	.002	-.015	.002	.110	.742	—	.110	.742
2	Span score	.011	-.022	.009	.559	.458	—	.334	.718
3	Conflict control, orienting	.018	-.051	.007	.191	.827	—	.258	.904
4	VSL accuracy, ASL accuracy	.037	-.068	.019	.555	.577	1.000	.354	.904

Table S10b. Final-model coefficients for GSI in SPL at Word 2.

Predictor	B	SE	*β*	95% CI for B	*t*	Unadjusted *p*	Holm-adjusted *p*	VIF	Tolerance
Midterm English score	1.214	2.427	.070	[-3.649, 6.077]	.500	.619	—	1.106	.905
Span score	-0.326	1.362	-.035	[-3.055, 2.403]	-.239	.812	—	1.190	.840
Conflict control	0.320	0.558	.087	[-0.799, 1.438]	.573	.569	—	1.313	.762
Orienting	-0.503	0.883	-.088	[-2.273, 1.268]	-.569	.572	—	1.358	.736
VSL accuracy	44.829	163.192	.037	[-282.215, 371.873]	.275	.785	1.000	1.053	.950
ASL accuracy	-128.502	124.901	-.151	[-378.808, 121.804]	-1.029	.308	1.000	1.223	.818

Table S11a. Model summary for predicting GSI in SPL at Word 3.

Step	Predictors entered	R^2	Adjusted R^2	ΔR^2	ΔF	Unadjusted p for ΔF	Holm-adjusted p	Model F	Model p
1	Midterm English score	.008	-.008	.008	.495	.485	—	.495	.485
2	Span score	.057	.025	.049	3.052	.086	—	1.782	.177
3	Conflict control, orienting	.063	-.003	.006	.181	.835	—	.957	.438
4	VSL accuracy, ASL accuracy	.079	-.022	.016	.468	.629	1.000	.782	.588

Table S11b. Final-model coefficients for GSI in SPL at Word 3.

Predictor	B	SE	β	95% CI for B	t	Unadjusted p	Holm-adjusted p	VIF	Tolerance
Midterm English score	-0.844	2.657	-.043	[-6.169, 4.481]	-.318	.752	—	1.106	.905
Span score	-2.157	1.491	-.204	[-5.145, 0.831]	-1.447	.154	—	1.190	.840
Conflict control	-0.173	0.611	-.042	[-1.397, 1.052]	-.283	.779	—	1.313	.762
Orienting	-0.438	0.967	-.068	[-2.376, 1.501]	-.452	.653	—	1.358	.736
VSL accuracy	-160.466	178.689	-.119	[-518.566, 197.634]	-.898	.373	1.000	1.053	.950
ASL accuracy	-43.639	136.761	-.046	[-317.715, 230.436]	-.319	.751	1.000	1.223	.818

*Note.*CI = confidence interval; VIF = variance inflation factor.
