# Supplementary material for: The Contributions of Statistical Learning to L2 Morphosyntax Processing Across Visual and Auditory Modalities in Chinese Adolescent Learners
Source: Behav Sci (Basel). 2026 Aug 21;16(8):1452. doi: 10.3390/bs16081452 (PMC13509579; doi:10.3390/bs16081452)
Supplement: Supplementary file 1 [file behavsci-16-01452-s001.zip › Supplementary File S5_ R Code and Random-Effects Simplification for the SPR and SPL Models.pdf]

Supplementary File S5. R Code and Random-Effects Simplification for the SPR and SPL Models

All models were fitted using lme4. Degrees of freedom and two-sided p values were obtained using lmerTest with the Satterthwaite approximation. Model-based comparisons were conducted using emmeans. Random effects were specified as uncorrelated, and singularity was evaluated using isSingular() with a tolerance of 10^{-4}.

```
# Linear mixed-effects model syntax
# Before running the code, import the item-audit worksheet as audit_items,
# the SPR trial-level data as spr_dat, and the SPL trial-level data as spl_dat.

library(dplyr)
library(tidyr)
library(stringr)
library(lme4)
library(lmerTest)
library(emmeans)

# ================================================================
# 1. Item exclusions for the primary and sensitivity analyses
# ================================================================

# Retain only the columns required for matching and exclusion.
audit_items <- audit_items[, c(1, 2, 3, 4, 6)]
names(audit_items) <- c(
  "Block", "ItemID", "GramSentence", "UngramSentence", "Decision"
)

audit_items <- audit_items %>%
  fill(Block, .direction = "down") %>%
  mutate(
    Item = paste(Block, ItemID, sep = "_"),
    Decision = str_squish(Decision)
  )

normalize_stimulus <- function(x) {
  x %>%
    as.character() %>%
    # Harmonize one transcription difference in the SPR dataset.
    str_replace_all(regex("\\btrid\\b", ignore_case = TRUE), "tried") %>%
    str_replace_all("/", " ") %>%
    str_replace_all("[?&+]", " ") %>%
    str_squish()
}
```

```
item_lookup <- audit_items %>%
  pivot_longer(
    c(GramSentence, UngramSentence),
    names_to = "Version",
    values_to = "Sentence"
  ) %>%
  mutate(StimulusKey = normalize_stimulus(Sentence)) %>%
  select(Item, Decision, StimulusKey) %>%
  distinct()

# Harmonize the original column names.
spr_dat <- spr_dat %>%
  rename(
    Grammaticality = Grammticity_All,
    SentenceOrg = Sentence
  )

spr_dat$StimulusKey <- normalize_stimulus(spr_dat$SentenceOrg)
spl_dat$StimulusKey <- normalize_stimulus(spl_dat$SentenceOrg)

spr_dat <- left_join(spr_dat, item_lookup, by = "StimulusKey")
spl_dat <- left_join(spl_dat, item_lookup, by = "StimulusKey")

stopifnot(!anyNA(spr_dat$Item), !anyNA(spl_dat$Item))

# Primary analysis: exclude only items classified as Exclude.
spr_primary <- spr_dat %>% filter(Decision != "Exclude")
spl_primary <- spl_dat %>% filter(Decision != "Exclude")

# Sensitivity analysis: additionally exclude items classified as
# Sensitivity Analysis; only Retain items remain.
spr_sensitivity <- spr_dat %>% filter(Decision == "Retain")
spl_sensitivity <- spl_dat %>% filter(Decision == "Retain")

# Verify the retained item and word-region counts.
c(
  SPR_primary_items = n_distinct(spr_primary$Item),
  SPR_primary_rows = nrow(spr_primary),
  SPR_sensitivity_items = n_distinct(spr_sensitivity$Item),
  SPR_sensitivity_rows = nrow(spr_sensitivity),
  SPL_primary_items = n_distinct(spl_primary$Item),
  SPL_primary_rows = nrow(spl_primary),
  SPL_sensitivity_items = n_distinct(spl_sensitivity$Item),
```

```
  SPL_sensitivity_rows = nrow(spl_sensitivity)
)

# ==================================================================
# 2. Variable coding
# ==================================================================

prepare_data <- function(dat) {
  dat %>%
    mutate(
      Subject = factor(Subject),
      Item = factor(Item),
      Grammaticality = factor(
        Grammaticality,
        levels = c("Gram", "Ungram")
      ),
      WordPosition = factor(
        WordPosition,
        levels = c("Word1", "Word2", "Word3")
      ),
      logRT = log(Word_RT),
      g = as.numeric(Grammaticality == "Ungram"),
      W2 = as.numeric(WordPosition == "Word2"),
      W3 = as.numeric(WordPosition == "Word3"),
      gW2 = g * W2,
      gW3 = g * W3
    )
}

spr_primary <- prepare_data(spr_primary)
spr_sensitivity <- prepare_data(spr_sensitivity)
spl_primary <- prepare_data(spl_primary)
spl_sensitivity <- prepare_data(spl_sensitivity)

model_control <- lmerControl(
  optimizer = "bobyqa",
  optCtrl = list(maxfun = 200000)
)

# ==================================================================
# 3. SPR models
# ==================================================================

# Initial SPR model used to evaluate the random-effects structure.
```

```
spr_m0 <- lmer(
  logRT ~ Grammaticality * WordPosition +
    (1 + g + W2 + W3 + gW2 + gW3 || Subject) +
    (1 + g + W2 + W3 + gW2 + gW3 || Item),
  data = spr_primary,
  REML = TRUE,
  control = model_control
)

isSingular(spr_m0, tol = 1e-4)

# Final nonsingular SPR structure.
spr_primary_model <- lmer(
  logRT ~ Grammaticality * WordPosition +
    (1 + g + W3 || Subject) +
    (1 + g + W2 + W3 || Item),
  data = spr_primary,
  REML = TRUE,
  control = model_control
)

spr_sensitivity_model <- update(
  spr_primary_model,
  data = spr_sensitivity
)

isSingular(spr_primary_model, tol = 1e-4)
isSingular(spr_sensitivity_model, tol = 1e-4)
summary(spr_primary_model, ddf = "Satterthwaite")
summary(spr_sensitivity_model, ddf = "Satterthwaite")
VarCorr(spr_primary_model)
VarCorr(spr_sensitivity_model)

# ============================================================
# 4. SPL models
# ============================================================

# Initial SPL model used to evaluate the random-effects structure.
spl_m0 <- lmer(
  logRT ~ Grammaticality * WordPosition +
    (1 + g + W2 + W3 + gW2 + gW3 || Subject) +
    (1 + g + W2 + W3 + gW2 + gW3 || Item),
  data = spl_primary,
  REML = TRUE,
```

```
  control = model_control
)

isSingular(spl_m0, tol = 1e-4)

# Final nonsingular SPL structure.
spl_primary_model <- lmer(
  logRT ~ Grammaticality * WordPosition +
    (1 + g || Subject) +
    (1 + g || Item),
  data = spl_primary,
  REML = TRUE,
  control = model_control
)

spl_sensitivity_model <- update(
  spl_primary_model,
  data = spl_sensitivity
)

isSingular(spl_primary_model, tol = 1e-4)
isSingular(spl_sensitivity_model, tol = 1e-4)
summary(spl_primary_model, ddf = "Satterthwaite")
summary(spl_sensitivity_model, ddf = "Satterthwaite")
VarCorr(spl_primary_model)
VarCorr(spl_sensitivity_model)

# ============================================================
# 5. Word-position-specific grammaticality comparisons
# ============================================================

region_comparisons <- function(model) {
  emm <- emmeans(
    model,
    ~ Grammaticality | WordPosition,
    lmer.df = "satterthwaite"
  )

  results <- as.data.frame(
    contrast(
      emm,
      method = list("Ungram - Gram" = c(-1, 1)),
      adjust = "none"
    )
```

```
  )

  results$p_holm <- p.adjust(results$p.value, method = "holm")
  results
}

spr_primary_results <- region_comparisons(spr_primary_model)
spr_sensitivity_results <- region_comparisons(spr_sensitivity_model)
spl_primary_results <- region_comparisons(spl_primary_model)
spl_sensitivity_results <- region_comparisons(spl_sensitivity_model)

spr_primary_results
spr_sensitivity_results
spl_primary_results
spl_sensitivity_results

sessionInfo()
packageVersion("lme4")
packageVersion("lmerTest")
packageVersion("emmeans")
```
