# Supplementary material for: The Contributions of Statistical Learning to L2 Morphosyntax Processing Across Visual and Auditory Modalities in Chinese Adolescent Learners
Source: Behav Sci (Basel). 2026 Aug 21;16(8):1452. doi: 10.3390/bs16081452 (PMC13509579; doi:10.3390/bs16081452)
Supplement: Supplementary file 1 [file behavsci-16-01452-s001.zip › Supplementary File S6_Complete Results of the Five Primary Hierarchical Regression Analyses.pdf]

Supplementary File S6. Complete Results of the Five Primary Hierarchical Regression Analyses

Table S12a. Model summary for predicting GSI in SPR at Word 1.

Step	Predictors entered	R^2	Adjusted R^2	ΔR^2	ΔF	Unadjusted *p* for ΔF	Holm-adjusted *p*	Model *F*	Model *p*
1	Midterm English score	.001	-.017	.001	.042	.839	—	.042	.839
2	Span score	.037	.004	.037	2.171	.146	—	1.107	.338
3	Conflict control, orienting	.104	.038	.066	2.031	.141	—	1.589 (4, 55)	.190
4	VSL accuracy, ASL accuracy	.147	.051	.044	1.362	.265	1.000	1.527 (6, 53)	.187

Note. N = 60; residual degree of freedom = 53 for all SPR model.

Table S12b. Final-model coefficients for GSI in SPR at Word 1.

Predictor	B	SE	*β*	95% CI for B	*t*	Unadjusted *p*	Holm-adjusted *p*	VIF	Tolerance
Midterm English score	-3.344	5.087	-.088	[-13.548, 6.860]	-.657	.514	—	1.127	.887
Span score	2.059	2.853	.101	[-3.663, 7.781]	.722	.474	—	1.219	.820
Conflict control	-2.030	1.148	-.258	[-4.334, .274]	-1.768	.083	—	1.323	.756
Orienting	3.561	1.817	.291	[-.082, 7.205]	1.960	.055	—	1.365	.733
VSL accuracy	291.428	262.472	.159	[-235.024, 817.880]	1.110	.272	1.000	1.282	.780
ASL accuracy	396.097	333.871	.155	[-273.564, 1065.758]	1.186	.241	1.000	1.056	.947

Note. CI = confidence interval; VIF = variance inflation factor.

Table S13a. Model summary for predicting GSI in SPR at Word 2.

Step	Predictors entered	R^2	Adjusted R^2	ΔR^2	ΔF	Unadjusted p for ΔF	Holm-adjusted p	Model F	Model p
1	Midterm English score	0.079	0.063	0.079	4.998	0.029	—	4.998	0.029
2	Span score	0.114	0.083	0.034	2.216	0.142	—	3.659	0.032
3	Conflict control, orienting	0.137	0.074	0.023	.740	0.482	—	2.183	0.083
4	VSL accuracy, ASL accuracy	0.142	0.045	0.005	.153	0.859	1	1.461	0.209

Table S13b. Final-model coefficients for GSI in SPR at Word 2.

Predictor	B	SE	β	95% CI for B	t	Unadjusted p	Holm-adjusted p	VIF	Tolerance
Midterm English score	9.034	4.347	.281	[.315, 17.754]	2.078	.043	—	1.127	.887
Span score	3.303	2.438	.190	[-1.586, 8.192]	1.355	.181	—	1.219	.820
Conflict control	-.773	.981	-.115	[-2.742, 1.195]	-.788	.434	—	1.323	.756
Orienting	1.806	1.552	.173	[-1.308, 4.919]	1.163	.250	—	1.365	.733
VSL accuracy	-82.163	224.284	-.053	[-532.021, 367.694]	-.366	.716	1.000	1.282	.780
ASL accuracy	121.272	285.296	.056	[-450.959, 693.503]	.425	.673	1.000	1.056	.947

Note. CI = confidence interval; VIF = variance inflation factor.

Table S14a. Model summary for predicting GSI in SPL at Word 1.

Step	Predictors entered	R^2	Adjusted R^2	ΔR^2	ΔF	Unadjusted p for ΔF	Holm-adjusted p	Model F	Model p

Step	Predictors entered	R^2	Adjusted R^2	ΔR^2	ΔF	Unadjusted *p* for ΔF	Holm-adjusted *p*	Model *F*	Model *p*
1	Midterm English score	0.032	0.015	0.032	1.957	0.167	—	1.957	0.167
2	Span score	0.043	0.011	0.012	.710	0.403	—	1.329	0.273
3	Conflict control, orienting	0.07	0.005	0.027	.835	0.439	—	1.078	0.376
4	VSL accuracy, ASL accuracy	0.091	-0.008	0.021	.639	0.532	1	.923	0.486

Note. N = 62; residual degree of freedom = 55 for all SPL model.

Table S14b. Final-model coefficients for GSI in SPL at Word 1.

Predictor	B	SE	β	95% CI for B	*t*	Unadjusted *p*	Holm-adjusted *p*	VIF	Tolerance
Midterm English score	2.351	2.291	0.139	[-2.240, 6.942]	1.026	0.309	—	1.106	0.905
Span score	0.538	1.286	0.059	[-2.038, 3.114]	0.418	0.677	—	1.19	0.84
Conflict control	-0.539	0.527	-0.151	[-1.595, .516]	-1.024	0.311	—	1.313	0.762
Orienting	-0.195	0.834	-0.035	[-1.867, 1.476]	-0.234	0.816	—	1.358	0.736
VSL accuracy	109.508	117.92	0.132	[-126.808, 345.825]	0.929	0.357	1	1.223	0.818
ASL accuracy	-105.723	154.071	-0.091	[-414.488, 203.042]	-0.686	0.495	1	1.053	0.95

Note. CI = confidence interval; VIF = variance inflation factor.

Table S15a. Model summary for predicting GSI in SPL at Word 2.

Step	Predictors entered	R^2	Adjusted R^2	ΔR^2	ΔF	Unadjusted p for ΔF	Holm-adjusted p	Model F	Model p
1	Midterm English score	0.029	0.013	0.029	1.797	0.185	—	1.797	0.185
2	Span score	0.029	-0.004	0	0.003	0.954	—	0.885	0.418
3	Conflict control, orienting	0.065	0	0.036	1.101	0.34	—	0.994	0.418
4	VSL accuracy, ASL accuracy	0.099	0.001	0.034	1.042	0.36	1	1.011	0.428

Table S15b. Final-model coefficients for GSI in SPL at Word 2.

Predictor	B	SE	β	95% CI for B	t	Unadjusted p	Holm-adjusted p	VIF	Tolerance
Midterm English score	2.278	1.795	0.171	[-1.319, 5.875]	1.269	0.21	—	1.106	0.905
Span score	0.167	1.007	0.023	[-1.851, 2.186]	0.166	0.869	—	1.19	0.84
Conflict control	-0.012	0.413	-0.004	[-.839, .815]	-0.03	0.976	—	1.313	0.762
Orienting	-0.937	0.653	-0.214	[-2.247, .372]	-1.434	0.157	—	1.358	0.736
VSL accuracy	-1.673	92.385	-0.003	[-186.816, 183.470]	-0.018	0.986	1	1.223	0.818
ASL accuracy	-173.931	120.707	-0.189	[-415.834, 67.972]	-1.441	0.155	1	1.053	0.95

Note. CI = confidence interval; VIF = variance inflation factor.

Table S16a. Model summary for predicting GSI in SPL at Word 3.

Step	Predictors entered	R^2	Adjusted R^2	ΔR^2	ΔF	Unadjusted p for ΔF	Holm-adjusted p	Model F	Model p
1	Midterm English score	0.001	-0.015	0.001	.076	0.784	—	.076	0.784
2	Span score	0.035	0.003	0.034	2.086	0.154	—	1.081	0.346
3	Conflict control, orienting	0.067	0.002	0.032	.970	0.385	—	1.025	0.402
4	VSL accuracy, ASL accuracy	0.144	0.051	0.077	2.481	0.093	0.465	1.546	0.181

Table S16b. Final-model coefficients for GSI in SPL at Word 3.

Predictor	B	SE	β	95% CI for B	t	Unadjusted p	Holm-adjusted p	VIF	Tolerance
Midterm English score	1.26	2.092	0.079	[-2.931, 5.452]	0.603	0.549	—	1.106	0.905
Span score	-1.513	1.174	-0.175	[-3.865, .839]	-1.289	0.203	—	1.19	0.84
Conflict control	-0.074	0.481	-0.022	[-1.038, .890]	-0.154	0.878	—	1.313	0.762
Orienting	-1.07	0.761	-0.204	[-2.597, .456]	-1.406	0.165	—	1.358	0.736
VSL accuracy	2.941	107.655	0.004	[-212.805, 218.687]	0.027	0.978	1	1.223	0.818
ASL accuracy	-313.146	140.659	-0.285	[-595.034, -31.258]	-2.226	0.03	0.331	1.053	0.95

Note. CI = confidence interval; VIF = variance inflation factor.
