# Supplementary material for: The Contributions of Statistical Learning to L2 Morphosyntax Processing Across Visual and Auditory Modalities in Chinese Adolescent Learners
Source: Behav Sci (Basel). 2026 Aug 21;16(8):1452. doi: 10.3390/bs16081452 (PMC13509579; doi:10.3390/bs16081452)
Supplement: Supplementary file 1 [file behavsci-16-01452-s001.zip › Supplementary_File S1_Previous Studies of Statistical Learning and L2 Morphosyntax.pdf]

Supplementary File S1. Previous Studies of Statistical Learning and L2 Morphosyntax

Table S1. Summary of previous studies linking statistical learning to L2 morphosyntactic learning, knowledge, and online processing

Study	Participants and L1	L2 and learning context	Morphosyntactic outcome and task	SL measure	Main finding
Godfroid and Kim (2021)	131 adult L2 English speakers; mixed L1 backgrounds	English; mean residence approximately 41 months	Multiple morphological and syntactic structures; word monitoring, SPR, elicited imitation, oral production, and timed/untimed GJTs	ASRT, ASL, VSL, Tower of London	ASRT predicted timed accuracy-based measures but not RT-based measures; ASL and VSL showed moderate convergence
Granena (2013)	50 early and 50 late L2 Spanish learners; Chinese L1	Spanish; early and late acquisition contexts	Agreement and nonagreement structures; metalinguistic knowledge and word-monitoring tasks	LLAMA D and SRT	Sequence-learning measures were associated with agreement-related outcomes, but the pattern differed between early and late learners
Suzuki and DeKeyser (2015)	63 advanced L2 Japanese speakers; Chinese L1	Japanese; shorter- and longer-residence groups	Japanese particle-related structures; elicited imitation with word monitoring	SRT	The association between SRT and GSI was observed primarily in the longer-residence group
Suzuki and DeKeyser (2017)	100 advanced L2 Japanese speakers; Chinese L1	Japanese; immersion context	Japanese morphosyntactic structures; visual-world, word-monitoring, SPR, timed GJT, and SPOT tasks	SRT and LLAMA F	SRT showed a small association with the visual-world measure, while relationships varied across implicit-knowledge tasks

Suzuki, DeKeyser, and Huang (2023)	65 L2 English learners and 28 English controls; Mandarin L1 for L2 group	English; immersion context	Definiteness and mass/count distinctions; visual-world eye tracking	SRT, LLAMA F, and MLAT Part 4	SRT was associated with the overall eye-tracking measure and definiteness processing but not mass/count processing
Suzuki et al. (2023)	25 advanced L2 Japanese speakers and 21 Japanese controls; Mandarin L1 for L2 group	Japanese; immersion context	Japanese case-marking particles; word monitoring during fMRI	SRT and LLAMA F	GSI was positively associated with LLAMA F; behavioral and neural results did not show identical aptitude relationships
Pili-Moss et al. (2025)	77 secondary-school EFL learners; German L1; aged 12–13	Instructed English in Germany	English question formation; digital gap-filling tests and written production	VSL, backward digit span, and explicit visual sequence learning	SL interacted with WM in predicting posttest accuracy and written-task outcomes
