# Supplementary material for: The Contributions of Statistical Learning to L2 Morphosyntax Processing Across Visual and Auditory Modalities in Chinese Adolescent Learners
Source: Behav Sci (Basel). 2026 Aug 21;16(8):1452. doi: 10.3390/bs16081452 (PMC13509579; doi:10.3390/bs16081452)
Supplement: Supplementary file 1 [file behavsci-16-01452-s001.zip › Supplementary_File S2_Participant Demographic and Language-Background Characteristics.pdf]

Supplementary File S2. Participant Demographic and Language-Background Characteristics

Table S2. Demographic and language-background characteristics of the participants.

**Characteristic**	**Descriptive result**	**Valid *N***
Age, years	*M* = 13.25, *SD* = 0.56, range = 12–14	64
Gender	Male: 21 (31.8%); Female: 45 (68.2%)	66
Age of first English exposure, years	*M* = 7.41, *SD* = 2.21	64
Age of formal English instruction, years	*M* = 7.23, *SD* = 1.80	64
Duration of English learning, years	*M* = 6.68, *SD* = 1.77	65
Additional English training	Yes: 17 (26.2%); No: 48 (73.8%)	65
Previous visit to an English-speaking country	Yes: 0 (0%); No: 61 (100%)	61
Regional dialect reported as part of L1 background	Yes: 18 (30.0%); No: 42 (70.0%)	60
Mandarin used at home	Yes: 44 (66.7%); No: 22 (33.3%)	66
Regional dialect used at home	Yes: 28 (42.4%); No: 38 (57.6%)	66
Literacy in another language	Yes: 5 (9.3%); No: 49 (90.7%)	54

Note. Valid N varies because some participants did not complete all items in the background questionnaire. Mandarin and regional-dialect use were not mutually exclusive. Dialect background was recorded as a binary variable and did not identify the specific dialect, proficiency, or frequency of use. Percentages were calculated using the valid responses for each variable.
